# Fluid Extract of Gelsenium

**Published:** 1874-12

**Authors:** 


*Fluid Extract of Gelseminum.*

Dr. J. C. Howard, of Florida, proposes for sale a fluid ext. of gelseminum which can be relied upon as genuine. Note hiscard and address in our advertising department. Give it a trial.

